# Universal Encryption of Individual Sequences Under Maximal Information Leakage

**DOI:** 10.3390/e27060551

**Published:** 2025-05-24

**Authors:** Neri Merhav

**Affiliations:** The Viterbi Faculty of Electrical and Computer Engineering, Technion—Israel Institute of Technology, Technion City, Haifa 3200003, Israel; merhav@technion.ac.il; Tel.: +972-4-8294737

**Keywords:** finite-state machine, Lempel–Ziv algorithm, maximal leakage, Shannon cipher system

## Abstract

We consider the Shannon cipher system in the framework of individual sequences and finite-state encrypters under the metric of maximal information leakage. A lower bound and an asymptotically matching upper bound on the leakage are derived, which lead to the conclusion that asymptotically minimum leakage can be attained by Lempel–Ziv compression followed by one-time pad encryption of the compressed bitstream.

## 1. Introduction

The information-theoretic approach that combines individual-sequence modeling with finite-state encoders and decoders has been extensively developed, representing a notable departure from the conventional reliance on probabilistic models traditionally used in source and channel modeling. This paradigm shift has gained traction across multiple areas of information theory, including lossless and lossy source coding [1,2,3,4,5,6]; source/channel simulation [7]; hypothesis testing [8,9]; prediction and decision making [10,11,12]; filtering [13]; and even error correction coding [14,15,16]. A concise overview of this expanding body of work can be found in [17], though these citations represent only a small fraction of the broader literature. By sharp contrast, the domain of information-theoretic security has remained largely anchored in probabilistic methods from Shannon’s foundational contributions [18] to more contemporary developments [19,20,21,22,23]. While these examples are far from exhaustive, they underscore the field’s persistent adherence to probabilistic frameworks.

To the author’s knowledge, only two significant departures from the dominant probabilistic approach in information-theoretic security exist: one is an unpublished technical report by Ziv [24] and the other is a subsequent development documented in [25]. In his report, Ziv proposes a novel framework in which the plaintext, destined for encryption using a secret key, is modeled as an arbitrary individual sequence. Within this setup, the encrypter functions as a general block encoder, while the eavesdropper is equipped with a finite-state machine (FSM) designed to distinguish between potential candidates for estimating the plaintext. A central aspect of Ziv’s model is the assumption that the eavesdropper possesses partial prior knowledge about the plaintext, formalized as a set of “acceptable messages”, which he defines as the acceptance set. Before intercepting the ciphertext, the eavesdropper’s uncertainty is characterized by the possibility that the plaintext could be any member of this set. Encryption is deemed perfectly secure if the ciphertext provides no additional information, i.e., if it does not reduce the size of the acceptance set, and thus renders the eavesdropper’s uncertainty unchanged. Accordingly, the size of the acceptance set quantifies uncertainty: the larger the set, the less the eavesdropper knows. The FSM attempts to rule out unacceptable sequences by testing them across different key sequences. Perfect secrecy is thus defined by the ciphertext’s inability to eliminate any members of the acceptance set. Ziv’s key result is that the minimum asymptotic key rate required for perfect secrecy, under this definition, is lower-bounded by the Lempel–Ziv (LZ) complexity of the plaintext sequence [6]. Furthermore, this lower bound is asymptotically tight. Perfect security can be achieved by applying a one-time pad (bitwise XOR with key bits) to the LZ-compressed version of the plaintext. This mirrors Shannon’s classical finding that the key rate must match the entropy rate of the source. More recent work [26] has expanded and sharpened Ziv’s original ideas in several important respects.

The subsequent work [25] offers a different take on the modeling approach and on achieving perfect secrecy for individual sequences. Instead of focusing on a finite-state eavesdropper with predefined knowledge, this approach models the encrypter itself as a finite-state machine (FSM), which processes both the plaintext and a stream of random key bits in a sequential manner. Central to this framework is the introduction of a new notion called finite-state encryptability, in the footsteps of finite-state compressibility introduced in [6]. Finite-state encryptability is defined as the minimum key rate required by any FSM-based encrypter to ensure that a particular measure of normalized empirical mutual information between the plaintext and ciphertext converges to zero as the block length increases. One of the main theoretical results in [25] asserts that the finite-state encryptability of a given individual sequence is lower-bounded by its finite-state compressibility. Stated differently, no finite-state encrypter can use a key rate below the sequence compressibility without compromising security, as defined in this setting. Once again, this lower bound is not merely theoretical; it can be asymptotically achieved through the same two-step process that was mentioned above: first compressing the plaintext using the Lempel–Ziv (LZ) algorithm and then applying one-time pad encryption to the resulting compressed bitstream. This mirrors earlier results in both compression and security, illustrating a deep connection between the two domains.

In this paper, we adopt the same model setting as in [25], but with a different security metric: the maximum leakage of information, which was first introduced by Issa, Wagner, and Kamath in [27] and then further explored in several more recent works, including [28,29,30,31,32], among others. This metric is closely related to, and similarly motivated by, the earlier security measure proposed in [33], which defines security as a scenario where the correct decoding exponent of the plaintext is not improved by the availability of the ciphertext, compared to that of blind guessing. For more details, see the last paragraph of Section 2.2.1. The maximum leakage metric is defined in a more general form and has a relatively straightforward expression, as demonstrated in [27] and further clarified in the following sections. As will be discussed in the sequel, the maximum leakage metric is particularly well suited for the individual-sequence setting considered here, as it is weakly dependent on the probability distribution of the plaintext, depending only on its support.

We derive both a lower bound and an asymptotically matching upper bound on the leakage, leading yet again to the conclusion that asymptotically optimal performance can be achieved by applying LZ compression followed by one-time pad encryption of the compressed bitstream. Thus, considering the above-mentioned earlier works, refs. [24,25,26], one of the messages of this work is that one-time pad encryption on top of LZ compression forms an asymptotically optimal cipher system from many aspects. Therefore, we believe that the deeper and more interesting contribution of this work is the converse theorem (Theorem 1 in the sequel) and its proof, asserting that the key rate that must be consumed to encrypt an individual sequence cannot be much smaller than the LZ complexity of the sequence minus the allowed normalized maximal information leakage.

This paper is structured as follows: In Section 2, we establish notation conventions, provide some necessary background, and formulate the problem studied in this work. In Section 3, we assert the main results and discuss them. Finally, in Section 4, we prove Theorem 1, which is the converse theorem.

## 2. Notation Conventions, Background, and Problem Formulation

### 2.1. Notation Conventions

In this paper, we adopt the following notation rules: Scalar random variables (RVs) are represented using uppercase letters, while their realizations (sample values) are denoted by the corresponding lowercase letters. The sets of possible values (alphabets) for these variables are indicated using calligraphic letters. This notation extends naturally to random vectors and their realizations. Specifically, an *n*-dimensional random vector will be denoted by appending a superscript indicating the dimension to the scalar symbol. For instance, $A^{n}$ (*n*–positive integer) refers to the random vector $\left(A_{1},\ldots ,A_{n}\right)$, and $a^{n}=\left(a_{1},\ldots ,a_{n}\right)$ denotes a particular instance of this vector, belonging to the set $A^{n}$, the *n*-fold Cartesian product of the alphabet A. Segment notations, such as $A_{i}^{j}$ and $a_{i}^{j}$, are used to represent substrings $\left(A_{i},\ldots ,A_{j}\right)$ and $\left(a_{i},\ldots ,a_{j}\right)$, respectively, for integers i≤j. When i=1, the index is dropped for brevity. If i>j, these notations are interpreted as representing the empty string. Additionally, for any real number *u*, the notation ${\left[u\right]}_{+}$ denotes max0, u. Unless otherwise noted, all logarithms and exponential functions throughout this paper are taken to base 2.

Throughout this article, information sources and channels will be generically represented by the letter *P*, following standard textbook notation. Subscripts will indicate the relevant random variables and any sort of conditioning, when applicable. For example, $P_{X^{n}}\left(x^{n}\right)$ denotes the probability mass function of the random vector $X^{n}$ evaluated at $x^{n}$, while $P_{Y^{n}|X^{n}}\left(y^{n}|x^{n}\right)$ represents the conditional probability of $Y^{n}=y^{n}$ given $X^{n}=x^{n}$. These subscripts may be omitted when the meaning is clear from context. Information-theoretic functionals such as entropy, mutual information, and related quantities will be expressed using standard symbols and conventions widely adopted in the information theory literature. In the remainder of this work, the symbol $x^{n}=\left(x_{1},\ldots ,x_{n}\right)$ will refer to a specific input sequence intended for encryption. Each element $x_{i}$, i=1,2,…,n, belongs to a finite input alphabet X, whose size is denoted by α.

### 2.2. Background

Before showing the main results and their proofs, let us revisit key terms and details related to the notion of maximal information leakage and the 1978 version of the LZ algorithm, also known as the LZ78 algorithm [6], which is the central building block in this work.

#### 2.2.1. Maximal Leakage of Information

As mentioned in the introduction, we adopt the maximal leakage [27] as our secrecy metric. For a probabilistic plaintext source, the maximal leakage from a secret random variable *X*, distributed according to $\left\{P_{X}\left(x\right),\;x\in X\right\}$, to another random variable *Y*, available to an adversary, and which is conditionally distributed given X=x according to $\left\{P_{Y|X}\left(y|x\right),\;x\in X,\;y\in Y\right\}$, is defined as

(1) $$L\left(X\to Y\right)\overset{▵}{=}\underset{U-X-Y-\hat{U}}{\sup}\log\frac{Pr\{\hat{U}=U\}}{\max_{u\in U}P_{U}\left(u\right)},$$

where the supremum is over all finite-alphabet random variables *U* and $\hat{U}$, with the Markov structure $U-X-Y-\hat{U}$. In other words, it is the maximum possible difference between the logarithm of the probability of guessing correctly some (possibly randomized) function of *X* based on *Y* and correctly guessing it blindly.

In Theorem 1 of [27], it was asserted and proved that the leakage can be calculated relatively easily using the following formula:

$$L\left(X\to Y\right)=\log\left[\sum_{y\in Y}\max_{\left\{x:\;P_{X}\left(x\right)>0\right\}}P_{Y|X}\left(y|x\right)\right].$$

Clearly, if $P_{Y|X}\left(y|x\right)$ is independent of *x* for all y∈Y, then L(X→Y)=0, which is the case of perfect secrecy. In general, the smaller the L(X→Y), the more secure the system is. In [27], it is shown that the maximal leakage has many interesting properties; one of them is that it satisfies a data processing inequality (see Lemma 1 of [27]). It is also shown in Section III of [27] that the maximal leakage has several additional operative meanings in addition to the original one explained above.

Note that the dependence on the distribution of the secret random variable, $P_{X}$, is rather weak, as it depends only on its support. When passing from single variables to vectors of length *n*, $L(X^{n}\to Y^{n})$ is defined in the same manner except that *x*, *y*, X, Y, $P_{X}\left(\cdot \right)$, and $P_{Y|X}\left(\cdot |\cdot \right)$ are replaced by $x^{n}$, $y^{n}$, $X^{n}$, $Y^{n}$, $P_{X^{n}}\left(\cdot \right)$, and $P_{Y^{n}|X^{n}}\left(\cdot |\cdot \right)$, respectively. In this case, the weak dependence of $L(X^{n}\to Y^{n})$ on $P_{X^{n}}$ makes it natural to use when $P_{X^{n}}$ is uncertain, or completely unknown, or even non-existent, such as in the individual sequence setting considered here. In this case, we adopt the simple definition

(2) $$L\left(x^{n}\to Y^{n}\right)\overset{▵}{=}\log\left[\sum_{y^{n}\in Y^{n}}\max_{x^{n}\in X^{n}}P_{Y^{n}|X^{n}}\left(y^{n}|x^{n}\right)\right],$$

corresponding to the full support $X^{n}$ for $x^{n}$, which accounts for a worst-case approach. The operational significance of maximal information leakage in our setting can then be understood in two ways: (i) Considering the definition (1), it allows arbitrary probability distributions (without any assumed structure) on $x^{n}$, including those that place almost all their mass on a single (unknown) arbitrary sequence, with regard to the individual-sequence setting considered here. (ii) Referring to Formula (2), it is evident that the leakage vanishes whenever $P_{Y^{n}|X^{n}}\left(y^{n}|x^{n}\right)$ is independent of $x^{n}$, which is an indisputable characterization for perfect secrecy in the individual-sequence setting too, where no distribution is assumed on $x^{n}$.

As mentioned in the introduction, a somewhat different security metric was proposed in [33], but it is intimately related to the maximal information leakage considered here. In [33], the idea was to define a system as secure if the probability of guessing *X* correctly is essentially the same if *Y* is present or absent. More precisely, if *X* and *Y* are random vectors of dimension *n*, then a system is considered secure if the correct decoding exponent of *X* in the presence of *Y* is the same as if *Y* is absent. Specifically, the correct decoding probability of *X* based on *Y* is

(3) $$P_{c}=\sum_{y}\max_{x}P_{XY}\left(x,y\right),$$

which is closely related to

(4) $$\begin{array}{ccc} 2^{L(X\to Y)} & = & \sum_{y}\max_{x}P_{Y|X}\left(y|x\right)\\ & = & \left|X\right|\cdot \sum_{y}\max_{x}\frac{P_{Y|X}\left(y|x\right)}{\left|X\right|}\\ & \overset{▵}{=} & \left|X\right|\cdot \sum_{y}\max_{x}P_{X}\left(x\right)P_{Y|X}\left(y|x\right)\\ & = & \frac{\sum_{y}\max_{x}P_{X}\left(x\right)P_{Y|X}\left(y|x\right)}{1/|X|}\\ & = & \frac{P_{c}^{i}}{P_{c}^{u}}, \end{array}$$

where $P_{X}\left(\cdot \right)$ is understood to designate the uniform distribution across X; accordingly, $P_{c}^{i}$ stands for the probability of correct decoding of a uniformly distributed *X* by an informed observer, namely one that has access to *Y*, whereas $P_{c}^{u}=1/\left|X\right|$ denotes the probability of correctly blind guessing the value of *X* (in the absence of *Y*).

#### 2.2.2. Lempel–Ziv Parsing

The incremental parsing process of the LZ78 algorithm is a sequential method applied to an input vector $x^{n}$ over a finite alphabet. In this process, each new phrase is the shortest substring that has not appeared before as a complete parsed phrase, except possibly for the final (incomplete) phrase. For instance, if one applies incremental parsing to the sequence $x^{15}=\mathrm{abbabaabbaaabaa}$, the outcome is a,b,ba,baa,bb,aa,ab,aa. Let $c(x^{n})$ designate the total number of phrases formed from $x^{n}$ by the incremental parsing process (in the example above, $c(x^{15})=8$). Also, let $LZ(x^{n})$ stand for the length of the LZ78 binary compressed representation for $x^{n}$. Theorem 2 of [6] easily leads to the following inequality:

(5) $$LZ\left(x^{n}\right)\leq c\left(x^{n}\right)\log c\left(x^{n}\right)+n\cdot \epsilon \left(n\right),$$

where ϵ(n) tends to zero as n→∞. In other words, the LZ code-length for $x^{n}$ cannot exceed an expression whose dominant term is $c\left(x^{n}\right)\log c\left(x^{n}\right)$. On the other hand, it turns out that $c\left(x^{n}\right)\log c\left(x^{n}\right)$ is also the dominant term of a lower bound (see Theorem 1 of [6]) to the minimum code-length attainable by any information lossless finite-state encoder with no more than *s* states, provided that $\log(s^{2})$ is very small compared to $\log c(x^{n})$. In view of these facts, we will be referring to $c\left(x^{n}\right)\log c\left(x^{n}\right)$ as the unnormalized *LZ complexity* of $x^{n}$, whereas the normalized LZ complexity will be defined as

(6) $$\rho_{\mathrm{LZ}}\left(x^{n}\right)\overset{▵}{=}\frac{c\left(x^{n}\right)\log c\left(x^{n}\right)}{n}.$$

### 2.3. Problem Formulation

Following the approach in [25], we adopt a finite-state model for encryption, described by the sextuple

E=(X,Y,Z,f,g,Δ),

where X is a finite input alphabet with cardinality α=|X|, Y is a finite collection of binary strings of variable length, possibly including the empty string λ (with zero length), Z is a finite set representing the internal states of the encrypter, $f:Z\times X\times {0,1}^{*}\to Y$ is the output function, g:Z×X→Z is the state transition function, and Δ:Z×X→0,1,2,… indicates the number of key bits used at each step. The encrypter *E* processes two infinite input sequences: a plaintext sequence $x=x_{1},x_{2},\ldots$, where each $x_{i}\in X$, and a key sequence $u=u_{1},u_{2},\ldots$, where each $u_{i}\in 0,1$. Given these inputs, the encrypter generates an infinite ciphertext sequence $y=y_{1},y_{2},\ldots$, with each $y_{i}\in Y$, while simultaneously transitioning through a corresponding state sequence $z=z_{1},z_{2},\ldots$, where each $z_{i}\in Z$. The evolution of these sequences is governed by a set of recursive equations, applied iteratively for each time step i=1,2,…

(7) $$\begin{array}{ccc} t_{i} & = & t_{i-1}+\Delta \left(z_{i},x_{i}\right),\;\;\;\;\;\;t_{0}\overset{▵}{=}0 \end{array}$$

(8) $$\begin{array}{ccc} k_{i} & = & \left(u_{t_{i-1}+1},u_{t_{i-1}+2},\ldots ,u_{t_{i}}\right) \end{array}$$

(9) $$\begin{array}{ccc} y_{i} & = & f(z_{i},x_{i},k_{i}) \end{array}$$

(10) $$\begin{array}{ccc} z_{i+1} & = & g(z_{i},x_{i}). \end{array}$$

Here, the initial state of the encrypter, $z_{1}$, is fixed and will be referred to as $z_{★}$ throughout. It is understood that when $\Delta (z_{i},x_{i})=0$, the encrypter uses no key bits at step *i*. In this case, the key fragment $k_{i}$ is defined to be the empty string λ. Similarly, if the output $y_{i}=\lambda$, then no output is generated at that step; the system effectively idles, meaning only the internal state changes in response to the input. More specifically, at each time step *i*, given the current state $z_{i}$ and the incoming plaintext symbol $x_{i}$, the encrypter consumes the next $\Delta (z_{i},x_{i})$ unused bits from the key sequence ***u*** to form $k_{i}$. It then produces an output $y_{i}$ (which may be empty) and transitions to the next state $z_{i+1}$ based on the function *g*. In summary, the operation at time *i* proceeds as follows: The encrypter is in state $z_{i}$, it receives input symbol $x_{i}$; it consumes $\Delta (z_{i},x_{i})$ key bits from ***u***, forming $k_{i}$; it generates output $y_{i}$; and it updates its state to $z_{i+1}$.

**Remark 1.**  *Note that the evolution of the state variable $z_{i}$ depends solely on the source inputs $\left\{x_{i}\right\}$ and is independent of the key bits. This design choice reflects the intended role of $z_{i}$, which is to retain memory of the source sequence $x^{n}$, allowing the encrypter to exploit empirical correlations and repetitive patterns within the plaintext. In contrast, maintaining memory of past key bits—which are assumed to be independent and identically distributed (i.i.d.)—offers no practical benefit and is therefore omitted. Moreover, the model can be naturally extended to include two state variables: one that evolves based only on the source sequence $\left\{x_{i}\right\}$ (as in the current setup) and another that evolves based on both $\left\{x_{i}\right\}$ and the consumed key bits $\left\{k_{i}\right\}$. In such a framework, the first state variable would continue to govern the update of the index $t_{i}$, while the second could influence the output function, allowing for more expressive or adaptive encryption mechanisms.*

An encrypter with *s* states, or an *s*-state encrypter, *E*, is one with |Z|=s. It is assumed that the plaintext sequence ***x*** is deterministic (i.e., an individual sequence), whereas the key sequence ***u*** is purely random, i.e., for every positive integer *n*, $P_{U^{n}}\left(u^{n}\right)=2^{-n}$.

A few additional notation conventions will be convenient: By $f(z_{i},x_{i}^{j},k_{i}^{j})$, (i≤j) we refer to the vector $y_{i}^{j}$ produced by *E* in response to the inputs $x_{i}^{j}$ and $k_{i}^{j}$ when the initial state is $z_{i}$. Similarly, the notation $g(z_{i},x_{i}^{j})$ will mean that the state $z_{j+1}$ and $\Delta (z_{i},x_{i}^{j})$ will designate $\sum_{\ell =i}^{j}\Delta \left(z_{\ell },x_{\ell }\right)$ under the same circumstances.

As explained in Section 2.2, we adopt the maximal leakage of information as our security metric, given by

$$L\left(x^{n}\to Y^{n}\right)\overset{▵}{=}\log\left[\sum_{y^{n}}\max_{x^{n}\in X^{n}}P_{Y^{n}|X^{n}}\left(y^{n}|x^{n}\right)\right].$$

An encryption system *E* is said to be *perfectly secure* if for every positive integer *n*, $L(x^{n}\to Y^{n})=0$. If $L(x^{n}\to Y^{n})\to 0$ as n→∞, we say that the encryption system is asymptotically secure.

An encrypter is referred to as *information lossless* (IL) if for every $z_{i}\in Z$, every sufficiently large *n* and all pairs $\left(x_{i}^{i+n},k_{i}^{i+n}\right)$, the quadruple $\left(z_{i},k_{i}^{i+n},f\left(z_{i},x_{i}^{i+n},k_{i}^{i+n}\right),g\left(z_{i},x_{i}^{i+n}\right)\right)$ uniquely determines $x_{i}^{i+n}$. Given an encrypter *E* and an input string $x^{n}$, the encryption key rate of $x^{n}$ with respect to *E* is defined as

(11) $$\sigma_{E}\left(x^{n}\right)\overset{▵}{=}\frac{\ell (k^{n})}{n}=\frac{1}{n}\sum_{i=1}^{n}\ell \left(k_{i}\right),$$

where $\ell \left(k_{i}\right)=\Delta \left(z_{i},x_{i}\right)$ is the length of the binary string $k_{i}$ and $\ell \left(k^{n}\right)=\sum_{i=1}^{n}\ell \left(k_{i}\right)$ is the total length of $k^{n}$.

**Remark 2.**  *It is worth noting that the definition of information losslessness used here is more relaxed and, thus, more general than the one given in [6]. In [6], the requirement must hold for every positive integer n, whereas in the present context, it is only required to hold for all sufficiently large n. The absence of information losslessness in the stricter sense of [6] does not contradict the ability of the legitimate decoder to reconstruct the source. Rather, it implies that reconstructing $x^{n}$ may require more than just the tuple $\left(z_{i},y_{i}^{i+n},k_{i}^{i+n},z_{i+n+1}\right)$; for example, some additional data from times later than i+n+1 may be needed.*

The set of all perfectly secure, IL encrypters {E} with no more than *s* states will be denoted by E(s). The minimum of $\sigma_{E}\left(x^{n}\right)$ over all encrypters in E(s) will be denoted by $\sigma_{s}\left(x^{n}\right)$, i.e.,

(12) $$\sigma_{s}\left(x^{n}\right)=\min_{E\in E(s)}\sigma_{E}\left(x^{n}\right).$$

Finally, let

(13) $$\sigma_{s}\left(x\right)=\underset{n\to \infty }{lim sup}\sigma_{s}\left(x^{n}\right),$$

and define the *finite-state encryptability* of ***x*** as

(14) $$\sigma \left(x\right)=\lim_{s\to \infty }\sigma_{s}\left(x\right).$$

Our purpose is to characterize these quantities and to point out how they can be achieved in principle.

## 3. Main Results

Our converse theorem, whose proof appears in Section 4, is the following:

**Theorem 1.**  *For every information lossless encrypter E with no more than s states,*

(15) $$\begin{array}{ccc} \frac{L(x^{n}\to Y^{n})}{n} & \geq & [\max_{x^{n}\in X^{n}}\left\{\rho_{LZ}\left(x^{n}\right)-\sigma_{E}\left(x^{n}\right)\right\}-\\ & & \delta_{s}\left(n\right)-\frac{\left(\alpha s-1)\log(n+1\right)}{n}-\frac{\log s}{n}{]}_{+}, \end{array}$$

*where $\delta_{s}\left(n\right)\leq O\left(\frac{\log(\log n)}{\log n}\right)$ for every fixed s. Equivalently, if $0\leq L(x^{n}\to Y^{n})\leq n\lambda$ for some given constant λ≥0, then for every $x^{n}\in X^{n}$ and every information lossless encrypter E∈E(s),*

(16) $$\sigma_{E}\left(x^{n}\right)\geq \rho_{LZ}\left(x^{n}\right)-\lambda -\delta_{s}\left(n\right)-\frac{\left(\alpha s-1)\log(n+1\right)}{n}-\frac{\log s}{n}.$$

*As for achievability, consider first an arbitrary lossless compression scheme that compresses $x^{n}$ at a compression ratio of $\rho \left(x^{n}\right)=L\left(x^{n}\right)/n$ and then applies one-time pad encryption to ${\left[L\left(x^{n}\right)-n\lambda \right]}_{+}$ compressed bits. Let $y^{n}$ denote the resulting (partially) encrypted compressed representation of $x^{n}$. Since the ${\left[L\left(x^{n}\right)-n\lambda \right]}_{+}$ key bits are purely random, the probablity of any $y^{n}$ that can be obtained from some $x^{n}$ is exactly $2^{-{\left[L\left(x^{n}\right)-n\lambda \right]}_{+}}$ and zero if $y^{n}$ cannot be obtained from any $x^{n}$. In other words, $\max_{x^{n}}P_{Y^{n}|X^{n}}\left(y^{n}|x^{n}\right)\leq 2^{-{\left[L\left(x^{n}\right)-n\lambda \right]}_{+}}$. Obviously, the length of $y^{n}$, denoted as $L(y^{n})$, is equal to $L(x^{n})$. Therefore, by denoting $L_{\max}=\max_{x^{n}\in X^{n}}L\left(x^{n}\right)$, we have the following:*

(17) $$\begin{array}{ccc} \exp_{2}\left\{L\left(x^{n}\to y^{n}\right)\right\} & = & \sum_{y^{n}\in Y^{n}}\max_{x^{n}}P_{Y^{n}|X^{n}}\left(y^{n}|x^{n}\right)\\ & = & \sum_{\ell =1}^{L_{\max}}\sum_{\left\{y^{n}:\;L\left(y^{n}\right)=\ell \right\}}\max_{x^{n}}P_{Y^{n}|X^{n}}\left(y^{n}|x^{n}\right)\\ & \leq & \sum_{\ell =1}^{L_{\max}}\sum_{\left\{y^{n}:\;L\left(y^{n}\right)=\ell \right\}}2^{-{\left[\ell -n\lambda \right]}_{+}}\\ & \leq & \sum_{\ell =1}^{L_{\max}}2^{\ell }2^{-{\left[\ell -n\lambda \right]}_{+}}\\ & \leq & \sum_{\ell =1}^{L_{\max}}2^{\ell }2^{-(\ell -n\lambda )}\\ & = & L_{\max}\cdot 2^{n\lambda }, \end{array}$$

*and, therefore,*

(18) $$L\left(x^{n}\to y^{n}\right)\leq n\lambda +\log L_{\max}.$$

*If $L_{\max}=O\left(n\right)$, then the dominant term is clearly nλ.*

**Remark 3.**  *The condition that $L_{\max}=O\left(n\right)$ is always easy to satisfy via a minor modification of any given compression scheme (if it does not satisfy the condition in the first place). First, test whether $L\left(x^{n}\right)<\left\lceil n\log\alpha \right\rceil$ or $L\left(x^{n}\right)\geq \left\lceil n\log\alpha \right\rceil$. If $L\left(x^{n}\right)<\left\lceil n\log\alpha \right\rceil$, add a header bit ‘0’ before the compressed representation of $x^{n}$; otherwise, add a header bit ‘1’ and then add the uncompressed binary representation of $x^{n}$ using ⌈nlogα⌉ bits. The resulting code-length would then be $L^{\prime }\left(x^{n}\right)=\min\left\{L\left(x^{n}\right),\left\lceil n\log\alpha \right\rceil \right\}+1$ bits.*

If the compression scheme is chosen to be the LZ78 algorithm, then

(19) $$\sigma_{E}\left(x^{n}\right)\leq \rho_{\mathrm{LZ}}\left(x^{n}\right)-\lambda +O\left(\frac{\log\log n}{\log n}\right),$$

which essentially meets the converse bound (16). We have therefore proved the following direct theorem:

**Theorem 2.**  *Given λ≥0, there exists a universal encrypter that satisfies*

(20) $$L\left(x^{n}\to Y^{n}\right)\leq n\lambda +\log n+O\left(1\right),$$

*and for every $x^{n}\in X^{n}$,*

(21) $$\sigma_{E}\left(x^{n}\right)\leq \rho_{LZ}\left(x^{n}\right)-\lambda +O\left(\frac{\log\log n}{\log n}\right).$$

**Discussion.**

A few comments are now in order.

1. We established both a lower bound and an asymptotically matching upper bound on the information leakage, leading once again to the conclusion that asymptotically optimal performance can be achieved by applying Lempel–Ziv (LZ) compression followed by one-time pad encryption of the compressed bitstream. As discussed in the introduction, together with earlier works, such as [24,25,26], this reinforces the message that one-time pad encryption applied after LZ compression yields an asymptotically optimal cipher system in several important respects. That said, we believe the deeper and more significant contribution of this work lies in the converse theorem (Theorem 1), which shows that the key rate required to securely encrypt an individual sequence cannot be substantially smaller than its LZ complexity minus the permitted normalized maximal information leakage, no matter what encryption strategy is employed.
2. Similarly as in [6], there is formally a certain gap between the converse theorem and the achievability scheme in its basic form, when examined from the viewpoint of the number of states, *s*, relative to *n*. While *s* should be small relative to *n* for the lower bound to be essentially $\rho_{\mathrm{LZ}}\left(x^{n}\right)$ (see Section 2.2 above), the number of states actually needed to implement LZ78 compression for a sequence of length *n* is basically exponential in *n*. In [6], the gap is closed in the limit of s→∞ (after taking the limit n→∞) by subdividing the sequence into blocks and restarting the LZ algorithm at the beginning of every block. A similar comment applies here too in the double limit of achieving σ(x).
3. As discussed in [26] in a somewhat different context, for an alternative to the use of the LZ78 algorithm, it can be shown that asymptotically optimum performance can also be attained by a universal compression scheme for the class of *k*-th order Markov sources, where *k* is chosen to be sufficiently large. In this case, $\rho_{\mathrm{LZ}}\left(x^{n}\right)$ in Theorems 1 and 2 should be replaced by the *k*-th order empirical entropy of order *k*, and some redundancy terms should be modified. However, one of these redundancy terms is $\frac{\log s}{k+1}$, which means that in order to compete with the best encrypter with *s* states, *k* must be chosen to be significantly larger than logs, so as to make this term reasonably small.
4. It is speculated that it may not be difficult to extend our findings in several directions, including lossy reconstruction, the presence of side information at either parties, the combination of both, and successive refinement systems in accordance to [32]. Other potentially interesting extensions are in broadening the scope of the FSM model to larger classes of machines, including FSMs with counters, shift-register machines with counters, and periodically time-varying FSMs with counters, as was carried out in Section III of [26]. Research in some of these directions will be explored in future studies.

## 4. Proof of Theorem 1

First, observe that

(22) $$\sigma_{E}\left(x^{n}\right)=\frac{1}{n}\sum_{i=1}^{n}\Delta \left(z_{i},x_{i}\right)=\sum_{x,z}\hat{P}\left(x,z\right)\Delta \left(z,x\right),$$

where $\hat{P}=\left\{\hat{P}\left(x,z\right),\;x\in X,\;z\in Z\right\}$ is the joint empirical distribution of (x,z) derived from $\left(x^{n},z^{n}\right)$. It is therefore seen that $\sigma_{E}\left(x^{n}\right)$ depends on $x^{n}$ only via $\hat{P}$. Accordingly, in the sequel, we will also use the alternative notation $\sigma_{E}\left(\hat{P}\right)$ when we wish to emphasize the dependence on $\hat{P}$. Let $T(x^{n})$ denote the set of ${\tilde{x}}^{n}\in X^{n}$, which, together with their associated state sequences, share the same empirical PMF $\hat{P}$ as that of $x^{n}$ along with its state sequence. Similarly as with $\sigma_{E}\left(\cdot \right)$, we also denote it by $T(\hat{P})$. In the sequel, we will make use of the inequality

(23) $$\frac{\log|T(x^{n})|}{n}\geq \rho_{LZ}\left(x^{n}\right)-\delta_{s}\left(n\right),$$

where $\delta_{s}\left(n\right)\to 0$ as n→∞ for a fixed *s* at the rate of $\frac{\log(\log n)}{\log n}$. The proof of Equation (23), which appears in various forms and variations in earlier papers (see, e.g., [34]), is provided in the Appendix A for the sake of completeness (see also the related Ziv’s inequality in Lemma 13.5.5 of [35]).

For later use, we also define the following sets:

(24) $$P\left(y^{n}\right)=\left\{\hat{P}:\;T\left(\hat{P}\right)\cap f^{-1}\left(y^{n}\right)\neq \emptyset \right\},$$

(25) $$Y\left(\hat{P}\right)=\left\{y^{n}:\;T\left(\hat{P}\right)\cap f^{-1}\left(y^{n}\right)\neq \emptyset \right\},$$

where

(26) $$\begin{array}{ccc} f^{-1}\left(y^{n}\right) & = & \{x^{n}:\;f\left(z_{★},x^{n},k^{n}\right)=y^{n}\\ & & \;\mathrm{for}\;\mathrm{some}\;k^{n}\in {\left\{0,1\right\}}^{n\sigma_{E}\left(x^{n}\right)}\}. \end{array}$$

In other words, $P(y^{n})$ is the set of all type classes of plaintext sequences for which some members can be mapped to $y^{n}$ by some key bit strings, whereas $Y(\hat{P})$ is the set of ciphertext sequences which can be obtained by some member of type class $T(\hat{P})$ and some key bit string. Now, observe that

(27) $$\begin{array}{ccc} \left|Y(\hat{P})\right| & \geq & |\{y^{n}:\;y^{n}=f\left(z_{★},x^{n},0^{n\sigma_{E}\left(x^{n}\right)}\right)\;\\ & & \mathrm{for}\;\mathrm{some}\;x^{n}\in T\left(\hat{P}\right)\}|\\ & \geq & \max_{z\in Z}|\{y^{n}:\;y^{n}=f\left(z_{★},x^{n},0^{n\sigma_{E}\left(x^{n}\right)}\right)\;\\ & & \mathrm{and}\;g\left(z_{★},x^{n}\right)=z\;\mathrm{for}\;\mathrm{some}\;x^{n}\in T\left(\hat{P}\right)\}|\\ & \overset{▵}{=} & \max_{z\in Z}\left|Y_{z}\left(\hat{P}\right)\right|\\ & \geq & \frac{\left|T(\hat{P})\right|}{s}, \end{array}$$

where the last inequality follows from the following consideration: Let $x^{n}$ exhaust all members of $T(\hat{P})$. For each such $x^{n}$, let $y^{n}=f\left(z_{★},x^{n},0^{n\sigma_{E}\left(x^{n}\right)}\right)$. Now, for every z∈Z, let $T_{z}\left(\hat{P}\right)$ denote the subset of $T(\hat{P})$ for which $z_{n+1}=g\left(z_{★},x^{n}\right)=z$, and we have already defined $Y_{z}\left(P\right)$ to denote the set of corresponding output sequences, $\left\{y^{n}\right\}$. Obviously, since ${\left\{T_{z}\left(\hat{P}\right)\right\}}_{z\in Z}$ form a partition of $T(\hat{P})$, then for some $z=z^{*}$, $|T_{z^{*}}\left(\hat{P}\right)|\geq |T\left(\hat{P}\right)|/s$. Therefore,

(28) $$\begin{array}{ccc} \max_{z}\left|Y_{z}\left(\hat{P}\right)\right| & \geq & |Y_{z^{*}}\left(\hat{P}\right)|\\ & = & |T_{z^{*}}\left(\hat{P}\right)|\\ & \geq & \frac{\left|T(\hat{P})\right|}{s}, \end{array}$$

where the equality is proved since the mapping between $x^{n}$ and $y^{n}$ is one-to-one given that $k^{n}=0^{n\sigma_{E}\left(x^{n}\right)}$, $z_{1}=z_{★}$, and $z_{n+1}=z^{*}$ by the information losslessness postulated, provided that *n* is sufficiently large as required. Now, let $Y_{n}^{+}$ denote that set of all $y^{n}\in Y^{n}$ for which $P_{Y^{n}|X^{n}}\left(y^{n}|x^{n}\right)>0$ for some $x^{n}\in X^{n}$. Then,

(29) $$\begin{array}{ccc} \exp_{2}\{L\left(x^{n}\to y^{n}\right\} & = & \sum_{y^{n}\in Y_{n}^{+}}\max_{x^{n}}P_{Y^{n}|X^{n}}\left(y^{n}|x^{n}\right)\\ & = & \sum_{y^{n}\in Y_{n}^{+}}\max_{x^{n}\in \phi^{-1}\left(y^{n}\right)}P_{Y^{n}|X^{n}}\left(y^{n}|x^{n}\right)\\ & = & \sum_{y^{n}\in Y_{n}^{+}}\max_{\hat{P}\in P\left(y^{n}\right)}\max_{x^{n}\in \phi^{-1}\left(y^{n}\right)\cap T\left(\hat{P}\right)}P_{Y^{n}|X^{n}}\left(y^{n}|x^{n}\right)\\ & \overset{\left(a\right)}{\geq } & \sum_{y^{n}\in Y_{n}^{+}}\max_{\hat{P}\in P\left(y^{n}\right)}\max_{x^{n}\in \phi^{-1}\left(y^{n}\right)\cap T\left(\hat{P}\right)}2^{-n\sigma_{E}\left(\hat{P}\right)}\\ & = & \sum_{y^{n}\in Y_{n}^{+}}\max_{\hat{P}\in P\left(y^{n}\right)}2^{-n\sigma_{E}\left(\hat{P}\right)}\\ & \geq & \frac{1}{M_{n}}\sum_{y^{n}\in Y_{n}^{+}}\sum_{\hat{P}\in P\left(y^{n}\right)}2^{-n\sigma_{E}\left(\hat{P}\right)}\\ & = & \frac{1}{M_{n}}\sum_{\hat{P}}\sum_{y^{n}\in Y\left(\hat{P}\right)}2^{-n\sigma_{E}\left(\hat{P}\right)}\\ & = & \frac{1}{M_{n}}\sum_{\hat{P}}\left|Y\left(\hat{P}\right)\right|\cdot 2^{-n\sigma_{E}\left(\hat{P}\right)}\\ & \overset{\left(b\right)}{\geq } & \frac{1}{M_{n}s}\sum_{\hat{P}}\left|T\left(\hat{P}\right)\right|\cdot 2^{-n\sigma_{E}\left(\hat{P}\right)}\\ & \geq & \frac{1}{M_{n}s}\cdot \max_{x^{n}\in X^{n}}\left|T\left(x^{n}\right)\right|\cdot 2^{-n\sigma_{E}\left(x^{n}\right)}\\ & \overset{\left(c\right)}{\geq } & \frac{1}{M_{n}s}\cdot \max_{x^{n}\in X^{n}}2^{n[\rho_{\mathrm{LZ}}\left(x^{n}\right)-\delta_{s}\left(n\right)]}\cdot 2^{-n\sigma_{E}\left(x^{n}\right)}\\ & = & \exp_{2}\{n\cdot \max_{x^{n}\in X^{n}}[\rho_{\mathrm{LZ}}\left(x^{n}\right)-\sigma_{E}\left(x^{n}\right)-\delta_{s}\left(n\right)-\\ & & \frac{\log M_{n}}{n}-\frac{\log s}{n}]\}\\ & \geq & \exp_{2}\{n\cdot \max_{x^{n}\in X^{n}}[\rho_{\mathrm{LZ}}\left(x^{n}\right)-\sigma_{E}\left(x^{n}\right)-\delta_{s}\left(n\right)-\\ & & \frac{\left(\alpha s-1)\log(n+1\right)}{n}-\frac{\log s}{n}]\}, \end{array}$$

where, in (a), we used the fact that $P_{Y^{n}|X^{n}}\left(y^{n}|x^{n}\right)>0$ implies $P_{Y^{n}|X^{n}}\left(y^{n}|x^{n}\right)\geq 2^{-n\sigma_{E}\left(x^{n}\right)}$ (because $P_{Y^{n}|X^{n}}\left(y^{n}|x^{n}\right)>0$ implies that there is at least one $k^{n}\in {\left\{0,1\right\}}^{n\sigma_{E}\left(x^{n}\right)}$ such that $f\left(z_{★},x^{n},k^{n}\right)=y^{n}$ and the probability of each such $k^{n}$ is $2^{-n\sigma_{E}\left(x^{n}\right)}$), and where $M_{n}$ is the number of different type classes, $\left\{\hat{P}\right\}$, which is upper-bounded by ${\left(n+1\right)}^{\alpha s-1}$. In (b), we used Equation (27); in (c), we used Equation (23). Finally, the operator ${\left[\cdot \right]}_{+}$ that appears in the assertion of Theorem 1 is due to the additional trivial lower bound $L(x^{n}\to Y^{n})\geq 0$. This completes the proof of Theorem 1.

## Data Availability

No new data were created or analyzed in this study. Data sharing is not applicable to this article.

## Appendix A. Proof of Equation (23)

Consider the LZ78 incremental parsing procedure applied to $x^{n}$ and let $c_{\ell zz^{\prime }}$, ℓ∈N, $z,z^{\prime }\in Z$, denote the number of phrases of length *ℓ*, which start at state *z* and end at state $z^{\prime }$. Clearly, $\sum_{\ell ,z,z^{\prime }}c_{\ell zz^{\prime }}=c\left(x^{n}\right)$, for which we will use the shorthand notation *c* in this appendix.

Given that $x^{n}\in T\left(\hat{P}\right)$, one can generate other members of $T(\hat{P})$ by permuting phrases of the same length that start at the same state and end at the same state. Thus, $|T\left(\hat{P}\right)|\geq \prod_{\ell ,z,z^{\prime }}\left(c_{\ell zz^{\prime }}!\right)$; thus,

(A1) $$\begin{array}{ccc} \log|T(\hat{P})| & \geq & \sum_{\ell ,z,z^{\prime }}\log\left(c_{\ell zz^{\prime }}!\right)\\ & \geq & \sum_{\ell ,z,z^{\prime }}c_{\ell zz^{\prime }}\log\frac{c_{\ell zz^{\prime }}}{e}\\ & = & \sum_{\ell ,z,z^{\prime }}c_{\ell zz^{\prime }}\log c_{\ell zz^{\prime }}-c\log e\\ & = & c\sum_{\ell ,z,z^{\prime }}\frac{c_{\ell zz^{\prime }}}{c}\left[\log\frac{c_{\ell zz^{\prime }}}{c}+\log c\right]-c\log e\\ & = & c\log c-cH(L,Z,Z^{\prime })-c\log e, \end{array}$$

where $H(L,Z,Z^{\prime })$ is the joint entropy of the auxiliary random variables *L*, *Z*, and $Z^{\prime }$, jointly distributed according to the distribution $\pi \left(\ell ,z,z^{\prime }\right)=c_{\ell zz^{\prime }}/c$, ℓ∈N, $z,z^{\prime }\in Z$. To further bound $\log|T(\hat{P})|$ from below, we now derive an upper bound to $H(L,Z,Z^{\prime })$:

(A2) $$\begin{array}{ccc} H(L,Z,Z^{\prime }) & \leq & H\left(L\right)+H\left(Z\right)+H\left(Z^{\prime }\right)\\ & \leq & H(L)+2\log s\\ & \leq & (1+EL)\log(1+EL)-(EL)\log(EL)+2\log s\\ & = & \left(1+\frac{n}{c}\right)\log\left(1+\frac{n}{c}\right)-\frac{n}{c}\log\frac{n}{c}+2\log s\\ & = & \frac{n}{c}\log\left(1+\frac{c}{n}\right)+\log\left(\frac{n}{c}+1\right)+2\log s\\ & \leq & \log\left(\frac{n}{c}+1\right)+\log\left(s^{2}e\right), \end{array}$$

where the third inequality is due to Lemma 13.5.4 of [35], the following equality is due to the relation $EL=\sum_{\ell ,z,z^{\prime }}\ell c_{\ell zz^{\prime }}/c=n/c$, and the last inequality is due to an application of the inequality log(1+u)≤uloge for all u>−1. It follows that

(A3) $$\begin{array}{ccc} \frac{\log|T(\hat{P})|}{n} & \geq & \rho_{\mathrm{LZ}}\left(x^{n}\right)-\frac{c}{n}\log\left(\frac{n}{c}+1\right)-\frac{c}{n}\log\left(s^{2}e\right)\\ & \geq & \rho_{\mathrm{LZ}}\left(x^{n}\right)-\frac{c}{n}\log\frac{n}{c}-\frac{c}{n}\log\left(1+\frac{c}{n}\right)-\frac{c}{n}\log\left(s^{2}e\right)\\ & \geq & \rho_{\mathrm{LZ}}\left(x^{n}\right)-\frac{c}{n}\log\frac{n}{c}-{\left(\frac{c}{n}\right)}^{2}\log e-\frac{c}{n}\log\left(s^{2}e\right)\\ & = & \rho_{\mathrm{LZ}}\left(x^{n}\right)-\delta_{s}\left(n\right), \end{array}$$

where

(A4) $$\delta_{s}\left(n\right)\overset{▵}{=}\frac{c}{n}\log\frac{n}{c}+{\left(\frac{c}{n}\right)}^{2}\log e+\frac{c}{n}\log\left(s^{2}e\right).$$

Since $\frac{c}{n}\leq \frac{\log\alpha }{\log n}\left(1+o\left(1\right)\right)$ (see Equation (6) of [6] and Lemma 13.5.3 of [35] and the reference therein), the second and the third terms of $\delta_{s}\left(n\right)$ are bounded by $O(1/\log^{2}n)$ and O(1/logn), respectively. The first term of $\delta_{s}\left(n\right)$ is upper-bounded by $O\left(\frac{\log(\log n)}{\log n}\right)$ (see Equation (13.124) of [35]).
